# Divorce prediction using machine learning algorithms in Ha’il region, KSA

**DOI:** 10.1038/s41598-023-50839-1

**Published:** 2024-01-04

**Authors:** Abdelkader Moumen, Ayesha Shafqat, Tariq Alraqad, Etaf Saleh Alshawarbeh, Hicham Saber, Ramsha Shafqat

**Affiliations:** 1 Department of Mathematics, College of Science, University of Ha’il, Ha’il, 55473 Saudi Arabia; 2 Department of Education, The University of Lahore, Sargodha, 40100 Pakistan; 3 Department of Mathematics and Statistics, The University of Lahore, Sargodha, 40100 Pakistan

**Keywords:** Psychology, Health care, Engineering, Mathematics and computing

## Abstract

The application of artificial intelligence (AI) in predictive analytics is growing in popularity. It has the power to offer ground-breaking solutions for a range of social problems and real world societal difficulties. It is helpful in addressing some of the social issues that today’s world seems incapable of solving. One of the most significant phenomena affecting people’s lives is divorce. The goal of this paper is to study the use of machine learning algorithms to determine the effectiveness of divorce predictor scale (DPS) and identify the reasons that usually lead to divorce in the scenario of Hail region, KSA. For this purpose, in this study, the DPS, based on Gottman couples therapy, was used to predict divorce by applying different machine learning algorithms. There were 54 items of the DPS used as features or attributes for data collection. In addition to the DPS, a personal information form was utilized to gather participants’ personal data in order to conduct this study in a more structured and traditional manner. Out of 148 participants 116 participants were married whereas 32 were divorced. With the use of algorithms artificial neural network (ANN), naïve bayes (NB), and random forest (RF), the effectiveness of DPS was examined in this study. The correlation based feature selection method was used to identify the top six features from the same dataset and the highest accuracy rate was 91.66% with RF. The results show that DPS can predict divorce. This scale can help family counselors and therapists in case formulation and intervention plan development process. Additionally, it may be argued that the Hail region, KSA sampling confirmed the Gottman couples treatment predictors.

## Introduction

Human relationships are the foundation of a civilized society. A family is a recognized group of people bound together by the bonds of marriage^1^. In married life, marriage separation or divorce can be the most unpleasant event which hurts members of the family and have negative affect on their life^2^. Divorce is one of the most critical phenomena impacting individuals’ lives as well as personal and social identity^3^. The rate of divorce in the Arab world has increased rapidly in recent years^4^. The rising divorce rate is a major problem in Saudi society because many couples consider it as the primary solution to end their struggles^5^. For a very long time, economists, psychologists, and sociologists have struggled with the important and difficult question of predicting people’s social preferences^6^. When scholars extract textual features from the content of online texts to support better user understandings and services, emotional signals and sentiment tendencies also draw more attention in the information age^7^. According to local media reports, divorce rate in Saudi Arabia has reached unprecedented levels in the last few years. In 2022, the average number of divorces is about 168 divorces per day (seven divorces per hour). There are three divorces for every ten marriages. According to Ministry of Justice administrative data, about 150,117 marriages and more than 57,500 divorces took place in 2020 (an increase of 8.9% and 12.7% respectively from 2019). The overall divorce rate for the total population reached 2.18 per 1000 population, an increase of 10.1% from 2019. Saudi Arabia’s population has grown by 13.8% since 2019, and the overall divorce rate per 1000 Saudi population reached 3.64%. The highest overall divorce rate among Saudis provinces was recorded in Hail (4.47%), followed by Northern Borders (4.42%). The lowest overall divorce rate was recorded in Jazan Province (2.50%), followed by Eastern Regions (2.84%), and Albaha (2.84%). Most of these divorces occurred in the last 3 months; October (14.6%), November (13.9%) and December (14.5%)^8,9^.

Yöntem and lhan^10^ built the DPS on the foundation of Gottman couples therapy which focused on divorce prediction. The Gottman couples therapy model explains the reasons that lead to divorce. John Gottman, a psychology professor at the University of Washington, created this technique. According to this method, the factors criticism, disdain, defensiveness, and obstructionism are identified as the four main causes of problems in a relationship. The strategy seeks to improve friendships by fostering constructive conflict resolution and a sense of purpose in life. This theory contains seven fundamental principles which are love maps, turning towards and discussing, positive perspective, solve problems together, managing conflicts and shared meaning^11^.

Determining divorce rates and identifying common causes of divorce usually help to reduce the rate of divorce cases. It also benefit family consultant and therapist when providing consultations to married couples and family members to help them in resolving their disputes.

The goal of this study is to use and compare the machine learning algorithms to determine the divorce success rate of DPS and identify the reasons that usually lead to divorce in the scenario of Ha’il region, KSA. For this purpose, the algorithms of ANN, NB, and RF were used to determine the success rate of DPS in the scenario of Ha’il Region, KSA. These three machine learning algorithms were applied and compared to determine the success rate of DPS, to predict divorce among Saudi couples, and to identify the reasons behind divorce.

## Related work

In order for computation equipment to be seamlessly integrated into people’s lives and to deliver more intelligent universal services through real-time sensing and dynamic interaction with the physical world, people want to closely relate the virtual world created by computation facilities to the physical world^12^. Researchers are continually tweaking the algorithms to improve their performance due to issues like the classifier performance declining with emotion refinement, the lack of a connection between sentences and the entire text, and the recognition of complex human emotions^13^. ext emotion analysis has grown in importance as one of the key areas of study in the field of natural language processing in recent years. It has been highlighted how to computationally identify and classify the opinions expressed in a piece of writing^6^. A variety of pattern recognition algorithms that were previously prohibitively expensive can now be used to uncover hidden values in large datasets thanks to advancements in computing technology^14^. Learning new ideas improves a person’s meta data and aids in the evaluation of individual class predictions by the local algorithms^15^. Many different fields, including signal processing, data mining, communications, finance, bio-medicine and robotics, etc. have heavily incorporated machine learning^16,17^.

Yöntem and lhan built the DPS on the foundation of Gottman couples therapy which focused on divorce prediction^10^. The Gottman couples therapy model, which was based on actual research, explained the most common reasons that lead to divorce. Within this paradigm, significant divorce predictors include the standards outlined in the Sound Relationship House concept. In this model, Gottman characterized four communication styles namely, Criticism, Contempt, Stonewalling, and Defensiveness, which can predict the end of a relationship^18^. Turkish researchers (Mustafa Kemal Yontem, Kemal Adem, Tahsin Lhan, and Serhat Kilicarslan) looked at divorce prediction from Turkish perspective^19,10^. Based on Gottman’s theory of couples, they created the DPS. They used ANN and relationship-based component determination. The Radial Basis Function neural network (RBF), ANN, and RF all achieved prediction rates of 97.64%, which was the highest. However, after selecting relationship-based highlights, they had 98.82% support for ANN. Furthermore, the accomplishment proportion was 97.64% using RBF and RF. Thus, they obtained the greatest results when they used the ANN model in conjunction with relationship-based element determination^20,21^.

Despite the lack of research on data mining techniques for divorce prediction, it is evident that various data mining techniques including classification, estimation and clustering are employed in numerous studies in the fields of psychology and psychiatry^10^. In 509 suicide attempters who were assessed in the emergency room, Baca-Garcia (2006) calculated the hospitalization choices of psychiatrists using data mining techniques. This study’s conclusions indicate that the Forward Selection approach has a 99% success rate in appropriately classifying patients^22^.

Song^23^ applied kNN, Bayes, and SVM data mining techniques to study psychological evaluation data of college students. Using SVM remarkable results were obtained regarding the binary classification model, with a success rate of 79.1%. Nguyen X^24^ employed data mining techniques to assess the effectiveness of insomnia symptoms in the management of long-term sleep apnea condition. Using decision trees, they showed that the unfavorable treatment responses were not related to long-term adjustment studies. A large number of radiology departments maintain an image database in an image archiving and communication system, which frequently offers a large number of examples for training neural networks. Since the 1960s, various computational methods for radiological diagnosis have been proposed and put into practise^16^. To improve the students’ operational effectiveness in the psychological data management system,

Qinghua^25^ implemented data mining technology based on the back-propagated ANN. The primary goal of this study is to avert psychological crises. Erikson et al.^10^ employed temporal data mining approaches to identify adverse medication responses. Rosenthal et al.^26^ utilized Data mining techniques to examine the variables influencing occupational results for people with mental impairments who received occupational rehabilitation services. They demonstrated that individuals getting job placement services have a favorable impact on occupational outcomes with the use of the CHAID algorithm^10^. Bae et al.^27^ implemented Decision tree algorithms to explore the factors that significantly affect the social functioning of schizophrenia patients^27^.

The development of the psychological equilibrium in society depends on healthy marriages. Researchers are seeking to counsel married couples on constructive marital remedies and disseminate information about tried-and-true methods. Research on the rehabilitation of patients who are hospitalized after suicidal attempts, recognizing the challenging parts for psycho-educational couples, and even the anticipated components of social functioning are now receiving more attention^10^.

In this study, the algorithms of ANN, NB, and RF were used to determine the success rate of DPS in the scenario of Ha’il Region, KSA. For this purpose three machine learning algorithms were applied and compared to determine the success rate of DPS, predict divorce among Saudi couples and to identify the reasons behind divorce. The prediction accuracy using ANN, NB, and RF was 80.00%, 85.00% and 90.00% respectively. However, after following the feature selection technique, the accuracy rate of NB and RF was increased to 88.14% and 91.66% respectively. The accuracy rate of ANN remained the same before and after the feature selection. Therefore, the best prediction was with RF after feature selection. The results show that DPS can predict divorce in the scenario of Ha’il region, KSA. This scale can help family counselors and therapists in case formulation and intervention plan development process. Additionally, it may be argued that the Ha’il region, KSA, sampling confirmed the Gottman couples treatment predictors.

## Methodology

### Study design and setting

**Figure 1:**
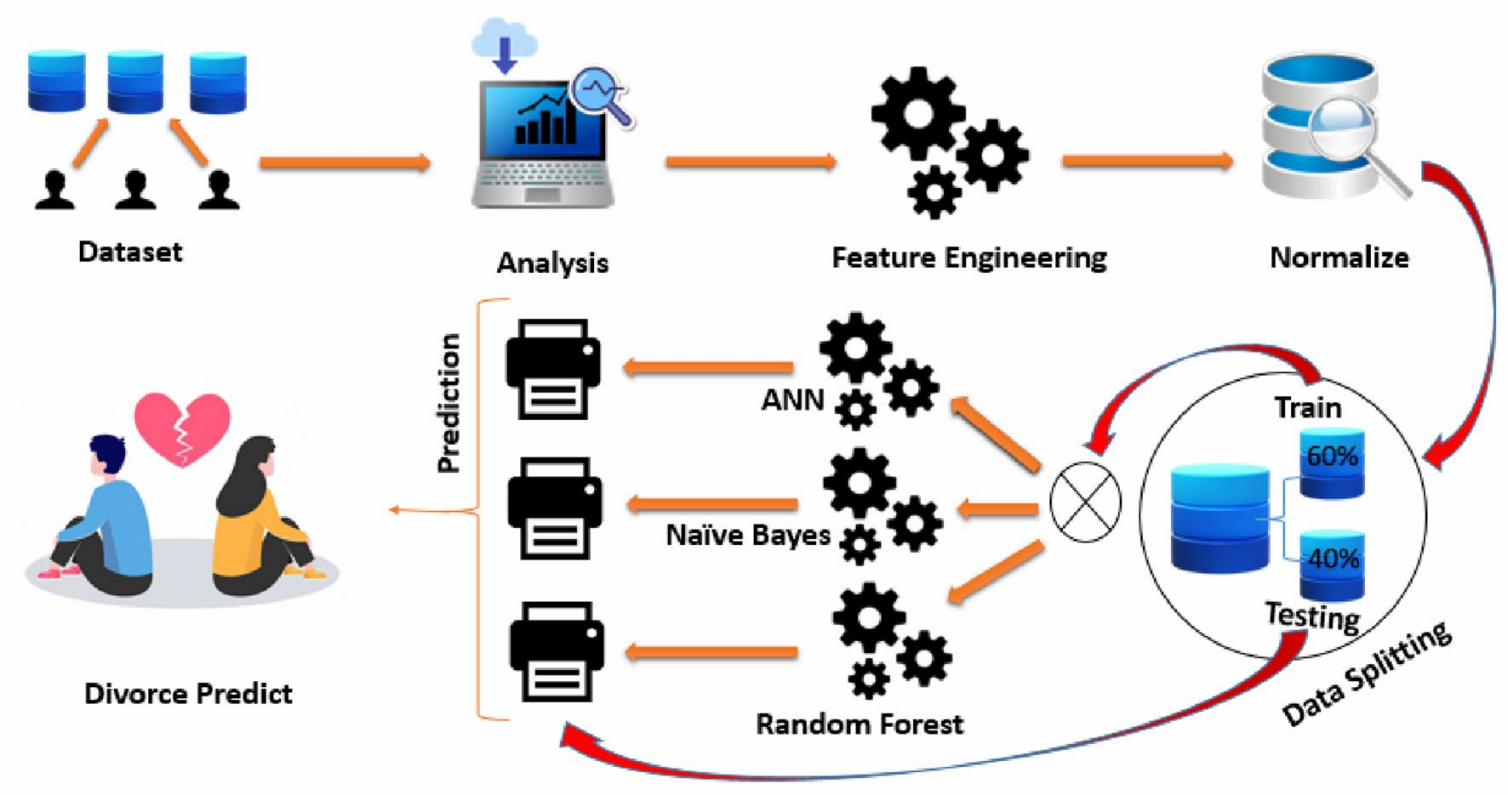
Study design^28^.

The nature of this study was descriptive and survey design was carried out to collect data from the participants. In order to collect data from the Ha’il region, KSA, convenient sampling technique was applied. A Google form was used to collect data from participant. The form was consisted of two parts. The first part of the form was about personal information; age, gender, educational background, monthly income, kind of marriage, and marital status. The second part consisted of 54 questions for DPS. The responses for 54 attributes were gathered on five point Likert scale (0 = Never, 1 = Rarely, 2 = Average, 3 = Often, 4 = Always). After data collection, the data were translated into English, and cleaning and preprocessing of data was performed. Then the algorithms of ANN, NB, and RF were used to determine the success rate of DPS (Fig. 1).

### Dataset description and participants

**Figure 2:**
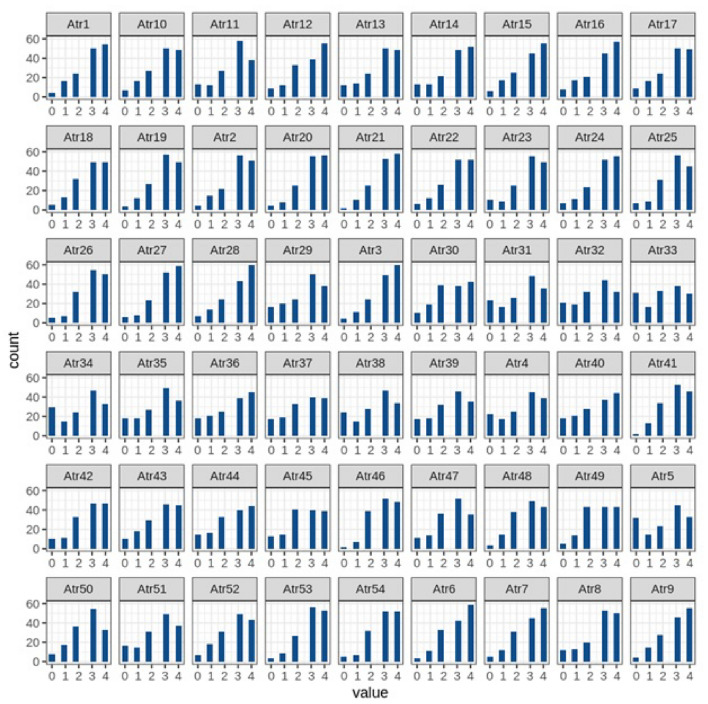
The divorce histogram analysis of all 54 attributes of DPS.

The dataset was consisted of 148 cases altogether. These cases were divided into two groups. One was training dataset with 60% cases and the other was testing dataset with 40% cases. At the end machine learning algorithms were applied, using Google Colab, twice before and after feature selection. Google Colab was also used to develop histogram analysis of all 54 attributes of DPS. Table 1 lists the 54 attributes and Fig. 2 shows histogram analysis.

**Table 1:** DPS attributes with detail^29^.

**No**	Names of attributes	Detail of attributes
1	Atr1	When I need it, I can take my discussions with my husband/wife from the beginning and correct it
2	Atr2	When I argue with my husband/wife, it will eventually work for me to contact him/her
3	Atr3	The time I spent with my husband/wife is special for me
4	Atr4	Rather than being family, we feel more like two strangers who share a space at home
5	Atr5	We do not have time at home as partners
6	Atr6	I enjoy my holidays with my husband/wife
7	Atr7	I enjoy traveling with my husband/wife
8	Atr8	Most of our goals are common
9	Atr9	I think that one day in the future, when I look back, I see that my spouse and I have been in harmony with each other
10	Atr10	When it comes to personal liberty, we both have similar beliefs
11	Atr11	We both have similar entertainment
12	Atr12	Most of our goals for people (children, friends, etc.) are the same
13	Atr13	Our dreams of living with each other are similar and harmonious
14	Atr14	We both are compatible with each other about what love should be
15	Atr15	In terms of living a good life, we both agree with each other
16	Atr16	Our views about the ideal marriage are similar
17	Atr17	We both agree on the roles that should be played in a marriage
18	Atr18	We both have similar values in trust
19	Atr19	I know exactly what my partner likes
20	Atr20	I know how my partner wants to be taken care of when he/she is sick
21	Atr21	I know my partner’s favorite food
22	Atr22	I can tell what kind of stress my partner is facing in his/her life
23	Atr23	I have knowledge of my partner’s inner world
24	Atr24	I know my partner’s basic concerns
25	Atr25	I know what my partner’s current sources of stress are
26	Atr26	I know my partner’s hopes and wishes
27	Atr27	I know my husband/wife very well
28	Atr28	I know my partner’s friends and his/her social relationships
29	Atr29	I feel aggressive when I argue with my husband/wife
30	Atr30	When discussing with my husband/wife, I usually use expressions such as“you always” or “you never”
31	Atr31	I can use negative statements about my partner’s personality during our discussions
32	Atr32	I can use offensive expressions during our discussions
33	Atr33	I can insult my partner during our discussions
34	Atr34	I can be humiliating when we argue
35	Atr35	My argument with my husband/wife is not calm
36	Atr36	I hate my partner’s way of opening a subject
37	Atr37	Our fights often occur suddenly
38	Atr38	I just start a fight with my husband/wife before I know what is going on
39	Atr39	When I talk to my husband/wife about something, my calm suddenly breaks
40	Atr40	When I argue with my husband/wife, I only go out and I do not say a word
41	Atr41	I am mostly stay silent to calm the environment a little bit
42	Atr42	Sometimes I think it is good for me to leave home for a while
43	Atr43	I would rather stay silent than argue with my husband/wife
44	Atr44	Even if I am right in the argument, I stay silent not to upset the other side
45	Atr45	When I argue with my husband/wife, I remain silent because I am afraid of not being able to control my anger
46	Atr46	I feel right in our discussions
47	Atr47	I have nothing to do with what I have been accused of
48	Atr48	I am not actually the one who is guilty about what I am accused of
49	Atr49	I am not the one who is wrong about problems at home
50	Atr50	I would not hesitate to tell my husband/wife about his/her inadequacy
51	Atr51	When I discuss it, I remind my husband/wife of his/her inadequacy
52	Atr52	I am not afraid to tell my husband/wife about his/her incompetence
53	Atr53	When one of us apologizes when our discussions go in a bad direction, the issue does not extend
54	Atr54	Even when things are challenging, I know we can put aside our disagreements

### Data processing, training, and test sets

Collected data contained some missing values. The missing values were filled with the mean value of the concerned feature. After processing, the data was divided into two parts. One part, which is the training dataset, consisted of about 60% of the total data and the other 40% form the testing dataset.

### Feature selection

Feature selection is the process of reducing the dimension of the data set through statistical techniques. In a nutshell, this process has the benefits of bettering mining performance, preventing overfitting of the algorithms, raising computational capacity, speeding up the data mining process, and improving understandability^30^. In this study, the six most useful features out of the 54 attributes that highly affect divorce were chosen by using CBFS (Correlation Based Feature Selection) approach. Correlation-based feature selection techniques, in supervised machine learning, chooses the optimal subset of features that comprises of characteristics which are substantially linked with the class but not with one another^31,32^. In this study, six attributes were obtained after applying CBFS (Correlation-based feature selection) technique on the dataset and their significant values were substantially linked with the class.

## Machine learning algorithms

In this section, discussion will be done about machine learning algorithms that were used to determine the success rate of DPS in the scenario of Ha’il Region, KSA. There were three machine learning algorithms applied and compared to determine the success rate of DPS, predict divorce among Saudi couples and to identify the reasons behind divorce. These three machine learning algorithms were ANN, NB and RF.

### Artificial neural network

In order to learn from data, generate new knowledge through learning and deal with an infinite number of variables, ANNs had been constructed. The ANN model was developed with the goal of simulating the human brain in a straightforward manner using computers. It focused on the mathematical modeling of biological neurons^10^.

The artificial neurons in this ANN algorithm are coupled to one another. A synthetic neuron is made up of four components. The dendrites transport the inputs from the sensory organs to the core in the human brain. The axons get the sum value that is produced by multiplying these input data by various weights. The synapses at the opposite end of the neuron get this value from the core via the axons, which then transmit it through the activation processes^33^.

In this study, the accuracy rate was 80.00% when ANN technique was simply used to the dataset. By using the same approach on the feature-selected dataset, the accuracy rate was remained same.

### Naïve bayes

NB^34,35^ is a probabilistic classifier that relies on the Bayes theorem and makes significant assumptions about the relationships between the features. The majority of applications that use NB computations include sentiment analysis, spam filtering, recommendation frameworks, etc. Although they are quick and easy to complete, their biggest obstacle is the need that features be provided without charge.

In this study, the accuracy percentage was 85.00% when the NB technique was used to analyze the dataset directly. However, while using the same technique on the feature-selected dataset, the accuracy rate increased to 88.33%.

### Random forest

The sacked group strategy known as RF relies on decision trees. When selecting a Random element, RF supports the differentiation of each tree separately. They then cast their votes in favor of the most prevalent class after it has produced many trees. Uneven information can be managed with the RF algorithm. It is rapid due to runtime and robust against over fitting^36^. After applying the RF method immediately to the dataset in this study, the obtained accuracy rate was 90.00%. But the accuracy rate proceeded from 90.00 to 91.66% by applying RF after feature selection.

### Correlation based feature selection

The six most useful features out of the 54 attributes that affect divorce were chosen using this approach. Correlation-based feature selection techniques, in supervised machine learning, chooses the optimal subset of features that comprises of characteristics which are substantially linked with the class but not with one another^31,32^. In this study, six attributes were obtained after applying CBFS (Correlation-based feature selection) technique on the dataset and their significant values were substantially linked with the class.

### Evaluation of models

Two metrics were used to evaluate the performance of the algorithms. The accuracy of the algorithms was calculated by applying the following metric^37^:

$$\begin{aligned} \text {Accuracy}=\frac{TP+TN}{TP+FP+FN+TN}. \end{aligned}$$

Kappa value of different algorithms was obtained by applying the following formula^38^:

$$\begin{aligned} k=\frac{p_o-p_e}{1-p_e}, \end{aligned}$$

where $p_o =$ Relative observed agreement among raters, $p_e =$ Hypothetical probability of chance agreement^38^.

## Hyperparameter tuning

Hyperparameter tuning was performed to improve the performance of applied machine learning algorithms. Table 2 represents the hyperparameters which were used in this study to improve the performance of applied machine learning algorithms regarding divorce prediction.

**Table 2:** Hyperparameters for machine learning algorithms.

Machine learning algorithms	Hyperparameters
ANN	batch_size=16, epochs=100, verbose=2, activation=’relu’
NB	Alpha (var_smoothing)= 1e−9, 1e−8, 1e−7
RF	n_estimators=100, random_state=42, cv=5, max_depth=5, verbose=2

## Results

In this study, probabilistic and ensemble learning classification algorithms along with ANNs were employed. As classifiers for this machine learning technique, ANN, NB, and RF have been used. Correlation-based feature selection (CBFS) technique was used for the feature selection portion. The feature vector is reduced to just six characteristics based on the identified correlation. The accuracy term was used to evaluate the algorithms. Every algorithm had been used twice, once with all features and once with only the chosen features. Google Colab was used to execute the machine learning algorithms. The computer contains a 4 GB RAM and an Intel Core i5-3320M, 2.60 GHz processor.

**Table 3:** Success rate for ANN.

Feature selection	Classification	No. of feature	Accuracy (%)	Kappa value
None	ANN	54	80.00	0.0000
CBFS	ANN	6	80.00	0.0000

The accuracy rate under ANN was the same with and without feature selection which was 80.00% (see Tables 3, 4).

**Table 4:** Confusion metrics for ANN.

Feature selection	Married	Divorced
None	0	12
	0	48
CBFS	0	12
	0	48

**Table 5:** Success rate for NB.

Feature selection	Classification	No. of feature	Accuracy (%)	Kappa value
None	NB	54	85.00	0.6154
CBFS	NB	6	88.33	0.6667

The accuracy rate was 85.00% with the direct application of NB, without feature selection, on dataset (Tables 5, 6). But the accuracy rate proceeded from 85.00 to 88.33% by applying NB after feature selection.

**Table 6:** Confusion metrics for NB.

Feature selection	Married	Divorced
None	11	1
	8	40
CBFS	10	2
	5	43

**Table 7:** Success rate for RF.

Feature selection	Classification	No. of feature	Accuracy (%)	Kappa value
None	RF	54	90.00	0.7059
CBFS	RF	6	91.66	0.7475

The accuracy rate was 90.00% with the direct application of RF, without feature selection, on dataset (Tables 7, 8). But the accuracy rate proceeded from 90.00 to 91.66% by applying RF after feature selection.

**Table 8:** Confusion metrics for RF.

Feature selection	Married	Divorced
None	10	2
	4	44
CBFS	10	2
	3	45

**Table 9:** Values of significance through CBFS.

Feature	Values of significance
Atr16	0.601
Atr15	0.589
Atr27	0.584
Atr20	0.584
Atr7	0.571
Atr3	0.570

**Figure 3:**
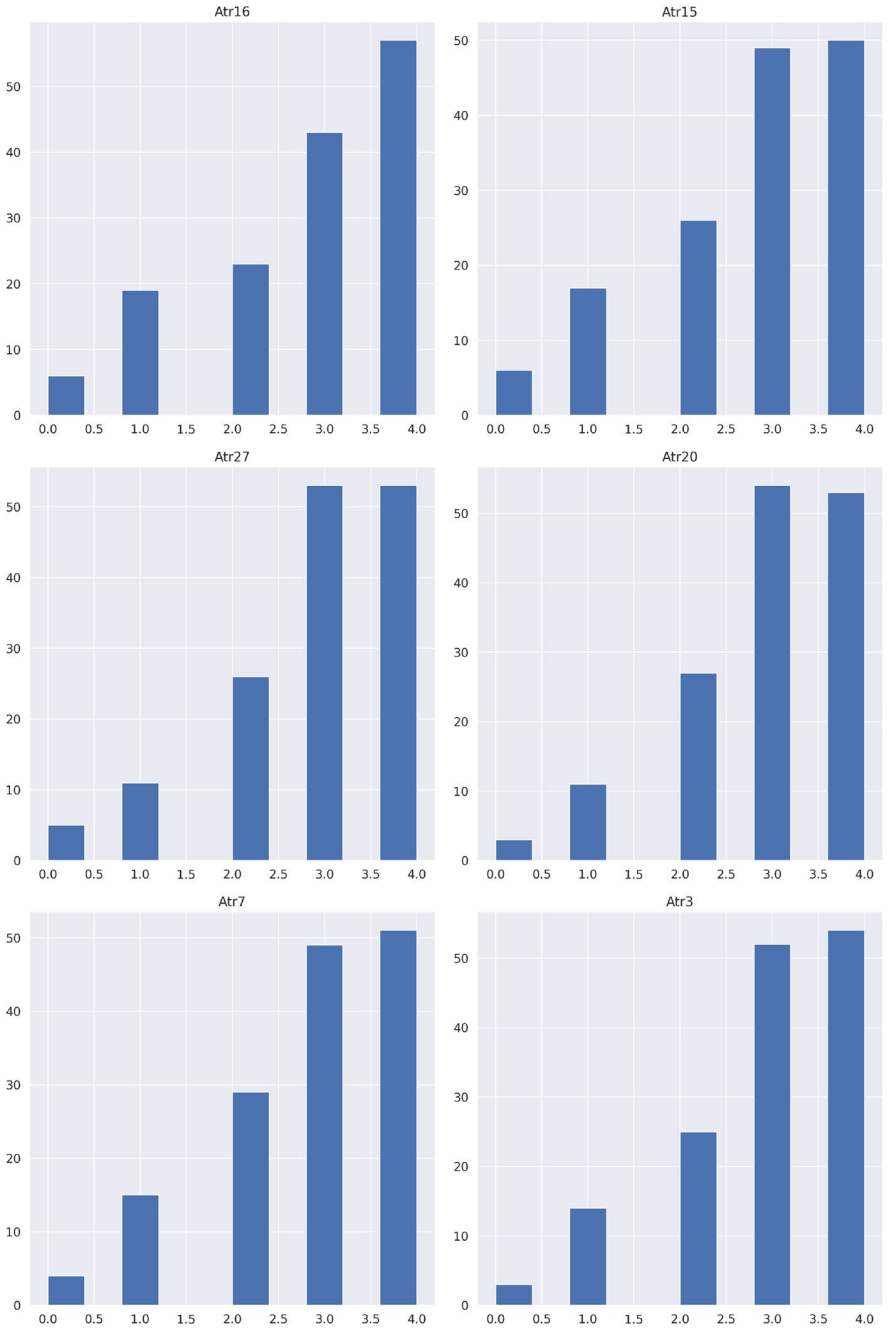
Divorce histogram analysis of top six highly effected features.

Table 9 lists the top six features and their significant values after using the correlation-based feature selection (CBFS) approach on the divorce dataset, Fig. 3 shows the analysis of these features. This indicates that Atr16 “Our views about the ideal marriage are similar”. was the highly affected feature. Other attributes include: Atr15 “In terms of living a good life, we both agree with each other”. Atr27 “I know my husband or wife very well”. Atr20 “I know how my partner wants to be taken care of when he/she is sick”. Atr7 “I enjoy traveling with my husband/wife”. Atr3 “The time I spent with my husband/wife is special for me”. These features were highly correlated and were used in the next phases. These DPS features might be helpful for counselors or therapists to make decisions in the course of their job.

**Table 10:** Classifiers performance with and without feature selection.

Feature selection	Classification	No. of feature	Accuracy (%)	Kappa value
None	ANN	54	80.00	0.0000
	NB		85.00	0.6154
	RF		90.00	0.7059
CBFS	ANN	6	80.00	0.0000
	NB		88.14	0.6667
	RF		91.66	0.7475

Table 10 shows that the highest accuracy rate was 90.00% under RF algorithm after the direct application of classification methods on divorce dataset. But after the selection of six most influential feature with the help of CBFS, the highest accuracy rate was 91.66% under RF. After analyzing the above results, it was observed that after applying different classification algorithms the most successful result was achieved through RF used with the combination of CBFS.

## Discussion

Turkish researchers (Mustafa Kemal Yontem, Kemal Adem, Tahsin Lhan, and Serhat Kilicarslan) looked at divorce prediction from Turkish perspective^19,10^. Based on Gottman’s theory of couples, they created the DPS. They used ANN and relationship-based component determination. The RBF, ANN, and RF all achieved prediction rates of 97.64%, which was the highest. However, after selecting relationship-based highlights, they had 98.82% support for ANN. Furthermore, the accomplishment proportion was 97.64 % using RBF and RF. Thus, they obtained the greatest results when they used the ANN model in conjunction with relationship-based element determination^20,21^. This study’s conclusions indicate that the Forward Selection approach has a 99 % success rate in appropriately classifying patients^22^. Song^23^ applied kNN, Bayes, and SVM data mining techniques to study psychological evaluation data of college students. Using SVM remarkable results were obtained regarding the binary classification model, with a success rate of 79.1%. But this study was conducted in the scenario of Ha’il Region, KSA. There were used the algorithms of ANN, NB, and RF to determine the success rate of DPS in the scenario of Ha’il Region, KSA. For this purpose three machine learning algorithms were applied and compared to determine the success rate of DPS, predict divorce among Saudi couples and to identify the reasons behind divorce. The prediction accuracy using ANN, NB, and RF was 80.00%, 85.00% and 90.00% respectively. However, after following the feature selection technique, the success rate for NB and RF was increased to 88.14% and 91.66% respectively but the success rate for ANN remained the same before and after feature selection. Therefore, the best prediction was with RF after feature selection. Thus our results aligns with the findings in Refs.^19,10^.

A strong output of the study is that DPS can predict divorce in the scenario of Ha’il region, KSA. Most likely a larger data set will support these finding. This scale can help family counselors and therapists in case formulation and intervention plan development process. Additionally, it may be argued that the Ha’il region, KSA, sampling confirmed the Gottman couples treatment predictors.

## Conclusion

According to the findings of this study, DPS can be helpful for divorce prediction. In order to find the machine learning algorithm with the greatest performance, the attempt was made to differentiate between regular features and selected features. In this study, there were applied three algorithms on the dataset. The accuracy rates for ANN, RF and NB were 80.00%, 88.14% and 91.66%, respectively. Therefore, the best prediction was with RF after feature selection. One of the objective of this study was to use machine learning algorithms to predict divorce rates among Hail region, KSA spouses. If an early detection mechanism can be put in place using the information presented in this research, it will prevent the dissolution of thousands of families. In order to utilize DPS in their screening procedures, this may be advantageous for ministries that have direct contact with families, such as the Ministry of Family and Social Affairs, the Ministry of National Education and the Ministry of Health. This scale can be used by the counseling services personnel to get to know the individual who will be receiving family counseling and family therapy. The formulation of the case and intervention strategy may be influenced by the scale’s results. Moreover, it may be argued that the Hail region, KSA sampling verified the divorce predictions from Gottman couples therapy. Further research should examine the effectiveness of the Gottman couples therapy model’s intervention strategies in the Hail region with the help of experimental research by creating psycho-educational programs based on Gottman couples therapy and by using numerous attribute selection techniques to locate connected or hyperactive attributes that best represent the Hail region, KSA perspective.

### Ethics statement

All subjects gave their informed consent for inclusion before they participated in the study. All methods were carried out in accordance with relevant guidelines and regulations. The informed consent was obtained from all subjects, and the protocol was approved by Research Deanship at University of Ha’il-Saudi Arabia number RD-21 067.

## Data Availability

Availability of supporting data. Data cannot be shared openly but are available on request from the corresponding author: Abdelkader Moumen @ mo.abdelkader@uoh.edu.sa.
