# Author Correction: Divorce prediction using machine learning algorithms in Ha’il region, KSA

**DOI:** 10.1038/s41598-024-53928-x

**Published:** 2024-03-05

**Authors:** Abdelkader Moumen, Ayesha Shafqat, Tariq Alraqad, Etaf Saleh Alshawarbeh, Hicham Saber, Ramsha Shafqat

**Affiliations:** 1 Department of Mathematics, College of Science, University of Ha’il, Ha’il, 55473 Saudi Arabia; 2 Department of Education, The University of Lahore, Sargodha, 40100 Pakistan; 3 Department of Mathematics and Statistics, The University of Lahore, Sargodha, 40100 Pakistan

Correction to: *Scientific Reports* 10.1038/s41598-023-50839-1, published online 04 January 2024

The original version of this Article contained an error in the Funding section where the project number was incorrect.

“This research has been funded by Scientific Research Deanship at University of Ha’il-Saudi Arabia through project number RG-21 067.”

now reads:

“This research has been funded by Scientific Research Deanship at University of Ha’il-Saudi Arabia through project number RD-21 067.”

The original Article has been corrected.

